# Use of Subfascial Passive Bile Bag Drainage for the Management of Durotomies in Spine Surgery

**DOI:** 10.7759/cureus.68397

**Published:** 2024-09-01

**Authors:** Luke Bauerle, Jeffrey E Wessell, Samantha Bindner, Brian F Saway, Laura Wolgamott, Stephen P Kalhorn

**Affiliations:** 1 College of Medicine, Medical University of South Carolina, Charleston, USA; 2 Department of Neurosurgery, Neurosurgical and Spine Institute of Savannah, Savannah, USA; 3 Department of Neurosurgery, Medical University of South Carolina, Charleston, USA; 4 Department of Neurosurgery, Stanford Health Care, Palo Alto, USA

**Keywords:** spine surgery, passive bile bag, subfascial drain, intentional durotomy, incidental durotomy, head-of-bed flat, durotomy repair, spinal csf leak

## Abstract

Introduction: Historically, the use of subfascial drains for the management of durotomies was avoided due to concerns about the creation of cerebrospinal fluid (CSF) fistulas. Currently, there are limited series utilizing subfascial drainage for CSF leak management, many of which utilize suction drainage. We report our experience with the use of subfascial passive drainage in the management of such leaks.

Objective: To demonstrate the efficacy of a passive subfascial bile bag for diversion of CSF post-operatively in concert with a post-operative head of bed (HOB) protocol for the management of durotomies in spine surgery.

Methods: We performed a retrospective chart review of patients who underwent spinal surgery at a single institution performed by one surgeon. Cases utilizing a passive subfascial bile bag for durotomies were identified. A total of 1,882 consecutive surgeries were reviewed, and 108 met the inclusion criteria. The primary outcome was return to the operating room (OR) and/or the need for lumbar drain placement. Patient sociodemographic information and pre-, intra-, and post-operative clinical characteristics were reviewed.

Results: A total of 108 patients underwent subfascial bile bag CSF diversion after intra-operative durotomy. Four patients (3.7%) experienced post-operative CSF leakage requiring lumbar drain placement, while only two (1.9%) patients required a return to the OR. One patient returned to the OR for symptomatic pseudomeningocele and the other for ongoing CSF drainage from their wound.

Conclusion: Durotomies are known to increase complication rates, including reoperation. The use of subfascial passive bile bag drainage in concert with a post-operative HOB protocol is a safe and effective manner to manage durotomies while minimizing the need for reoperation.

## Introduction

A durotomy-induced cerebrospinal fluid (CSF) leak, whether intentional or not, is one of the most frequently reported complications associated with spine surgery, with reported rates varying from 1.6% to 16% [1-10]. Numerous risk factors for incidental (unintentional) durotomies have been identified, including patient age, surgeon experience, lumbar surgery, pre-existing conditions, and revision surgery [1]. Intentional dural openings are required for the treatment of intradural pathology, and though these are more readily closed primarily than unintentional durotomies, both can lead to persistent CSF leaks. If not addressed, CSF leaks can result in a variety of complications, such as severe headache, cranial nerve deficits, symptomatic pseudomeningocele formation, nerve root entrapment, intracranial hemorrhage, meningeal pseudocyst formation, and dural cutaneous CSF fistulas leading to meningitis and arachnoiditis [8,11-15]. As a result, durotomies may necessitate additional surgery, impair patient quality of life, and increase healthcare-related costs for both the patient and the healthcare system [11].

Current recommendations for the prevention of CSF leak-related complications include bed rest with the head-of-bed (HOB) flat in addition to lumbar drain placement, though primary dural repair is widely considered to be the most effective way to prevent persistent CSF leakage [15-17]. However, in situations in which visualization and/or access to the durotomy is limited, traditional suturing may be technically difficult, if not impossible. Attempts to remove additional bone may not always be feasible and risk destabilization of the spine [11]. Subfascial drains providing continuous CSF removal have been explored as viable options for CSF diversion following a durotomy, though prior dogma relating to such drains has hindered its utilization due to concerns about the formation of CSF-fistulas and the potential exacerbation of CSF leaks [18]. Additionally, surgeons have recommended bed rest, with the HOB flat in an attempt to prevent post-operative complications after durotomy. The rationale is that a flat HOB reduces hydrostatic pressure at the site of the tear by Pascal’s principle, promoting healing and minimizing symptoms [19].

Though studies exploring the utilization of subfascial non-suction CSF drains in concert with traditional HOB flat post-operative management algorithms are present in current literature, these studies typically include small patient populations, or exclude multiple comorbid conditions, thus lowering their statistical power and generalizability. Therefore, further exploration of this approach to the management of subfascial diversion of CSF following spinal surgery in a larger patient population is needed. The purpose of this study was to demonstrate the safe and effective management of durotomies in spine surgery using a non-suction subfascial bile bag® (Becton, Dickinson and Company, Franklin Lakes, USA) for the diversion of CSF post-operatively in combination with a post-operative HOB flat management protocol, when appropriate.

This article was previously presented as a poster presentation at the 2024 South Carolina Medical Association Annual Meeting on April 27, 2024.

## Materials and methods

The study was designed as a retrospective, observational study consisting of patients aged 18 years or older who underwent spine surgery between August 2014 and November 2021. The study was approved by the Medical University of South Carolina’s Institutional Review Board (IRB) (Charleston, South Carolina). The requirement for informed patient consent for this study was waived because of the retrospective nature of the study and the minimal risk to patients in study involvement.

Potential participants were identified using the electronic health record and reviewed for eligibility. All enrolled patients had to fulfill eligibility criteria, with all selected study participants having to be 18 years of age or older at the time of their respective spinal procedure and who underwent passive bile bag drainage for the post-operative management of either an incidental or non-incidental durotomy sustained during their procedure. Following eligibility, all pertinent patient data was then de-identified for further analysis. Sociodemographic information (patient gender, smoking status, race, and reported comorbidities), pre-operative variables (pre-operative diagnosis, surgical indication, history of spine surgery), and intra-operative variables (operated spine levels, nature of durotomy, dural repair method, skin closure method) were all extracted from the electronic health record. Post-operative information including reoperation, surgical complications, length of stay, and readmission within 30 days were also collected, from which clinical outcomes were then compared based on operated spine levels, nature of durotomy (intentional or incidental), and method of dural and skin closure.

The surgeon’s practice was to only place non-suction bile bag drains for spinal procedures in the setting of a spinal fluid leak. All patients received antibiotic prophylaxis prior to surgery with a #7 Jackson-Pratt® drain catheter tip (Cardinal Health, Inc., Dublin, USA) placed strictly within the subfascial compartment. The drain catheter tip was always placed above the dural graft, adhesive, or sutures at the time of dural closure. The drain was connected to a bile bag and secured at the level of the incision, or in the case of an HOB flat, at the level of the bed. In cases of primary dural repair, running 4-0 Nurolon sutures (Ethicon, Somerville, USA) were routinely utilized. Regardless of the wound or dural closure method utilized for each patient, fascial and muscular closures were achieved with interrupted 0 Vicryl sutures (Ethicon, Somerville, USA). Subcutaneous closures were achieved with interrupted inverted 2-0 Vicryl sutures, with running nylon sutures utilized in cases of skin closure via traditional suturing. The drain exited the wound site with a tube diameter of 2.3 mm (0.092 inches) and was secured with 2-0 Vicryl sutures.

For post-operative management, the patient was on strict bed rest with no mobilization until deemed appropriate to mobilize based on daily CSF output. For patients with operated spine levels at or below T7, HOB was completely flat to allow for fascial healing, with HOB elevated to 30 degrees or less during meals only. All patients were started on an anticoagulation regimen of enoxaparin within 24 hours following their respective spinal procedures for deep vein thrombosis (DVT) prophylaxis, with no DVTs reported in our patient cohort. This protocol was continued until the CSF output was less than 40cc over a 24-hour period or after four full days of bed rest, regardless of drain output. No clamping test was conducted before complete drain removal. After drain removal and closure of the drain site using absorbable 2-0 Vicryl sutures, patients were mobilized without restrictions. Monitoring for the development of significant post-operative CSF leaks was continued up until the time of discharge.

Statistical analysis

All statistical analyses were conducted using Statistical Package for the Social Sciences (IBM Corp. Released 2021. IBM SPSS Statistics for Windows, Version 28.0. Armonk, NY: IBM Corp). Univariate analyses of sociodemographic and clinical characteristics were conducted. Categorical variables are listed as numbers (%) and were compared using the Chi-square test, unless expected cell counts were five or less, in which case the Fisher's exact test was used. Continuous variables were tested for normality using the Kolmogorov-Smirnov D statistic. Non-normally distributed variables are listed as median with interquartile range (IQR) and were analyzed using the Mann-Whitney U test. Statistical significance was defined as a p-value of less than 0.05.

## Results

**Figure 1:**
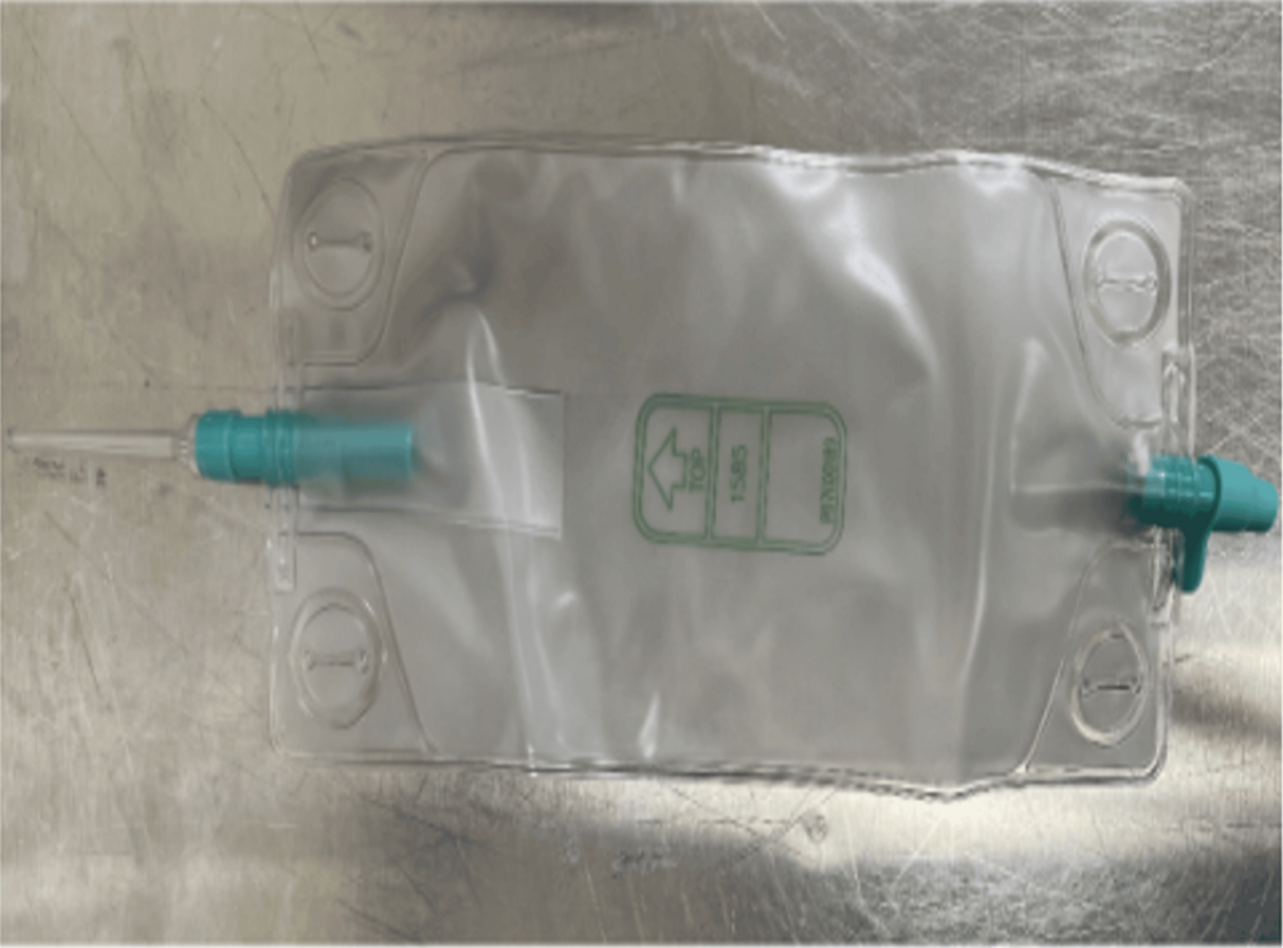
Example of a bile bag used

A total of 1,882 surgeries were identified and screened for usage of a bile bag (Figure 1). A total of 108 patients met the inclusion criteria of this study. Sociodemographic information is listed in Table 1. There was a slight female predominance with 56 (51.9%) included in the study. Fifteen of the 108 patients (13.9%) were current smokers and 38 (35.2%) had a prior history of smoking. Co-morbidities present in the study population included 31 (28.7%) subjects with diabetes, 65 (60.2%) having hypertension, and 17 (15.7%) having active corticosteroid use.

**Table 1:** Demographic characteristics for patients managed with passive subfascial bile bag for durotomy in spine surgery

Variable (N = 108)	n (%)
Gender	
Female	55 (50.9%)
Male	53 (49.1%)
Smoking Status	
Current Smoker	15 (13.9%)
Previous Smoker	31 (28.7%)
Race	
White	85 (78.7%)
Black or African-American	19 (17.6%)
Hispanic	2 (1.9%)
Other	2 (1.9%)
Comorbidities	
Type II Diabetes Mellitus	31 (28.7%)
Hypertension	65 (60.2%)
Congestive Heart Failure	4 (3.7%)
Prior Steroid Use	17 (15.7%)
Chronic Obstructive Pulmonary Disease	3 (2.8%)
Peripheral Vascular Disease	2 (1.8%)
Connective Tissue Disease	1 (0.9%)
Autoimmune Disease	5 (4.6%)
Vitamin Deficiency	2 (1.8%)

Reported pre-operative diagnoses included spinal stenosis, intradural tumor, and arachnoid web with 57 patients (52.8%), 28 patients (25.9%), and 17 patients (15.7%), respectively (Table 2). Indications for surgery included radiculopathy in 58 of the 108 subjects (53.7%), 45 (41.7%) for myelopathy, and five (4.6%) due to trauma-related instability. A prior history of spinal surgery was present in 40 patients (37%).

**Table 2:** Pre-operative characteristics for patients managed with passive subfascial bile bag for durotomy in spine surgery

Variable (N = 108)	n (%)
Pre-operative Diagnosis	
Intradural Tumor	28 (25.9%)
Arachnoid Web	17 (15.7%)
Spinal Stenosis	57 (52.8%)
Pseudomeningocele Repair	1 (0.9%)
Tethered Cord	2 (1.8%)
Post-operative Hematoma	1 (0.9%)
Fracture	2 (1.8%)
Indication for Surgery	
Radiculopathy	58 (53.7%)
Myelopathy	45 (41.7%)
Traumatic Instability	5 (4.6%)
History of Prior Spine Surgery	
Yes	40 (37%)
No	68 (63%)

Table 3 presents general intra-operative information relating to each spine surgery within the included patient cohort. Of the 108 subjects included in the study, 13 (12%) were isolated to the cervical spine, 52 (48.1%) were thoracic, 35 (32.4%) were lumbar, one (0.9%) cervicothoracic, and seven (6.5%) thoracolumbar. Unintentional durotomies occurred in 55 (50.9%) of the cases. Dural closure methods utilized included 56 (51.9%) with suture alone, 45 (41.7%) closed using either a suturable dural substitute or a muscle patch, and seven (6.5%) closed with fibrin glue alone. Regarding the wound closure method, 57 (52.8%) were closed with Dermabond or Prenio Dermabond (Ethicon, Somerville, USA), eight (7.4%) with sutures, and 43 (39.8%) with staples.

**Table 3:** Intra-operative characteristics for patients managed with passive subfascial bile bag for durotomy in spine surgery

Variable (N = 108)	n (%)
Level of Surgery	
Cervical	13 (12%)
Thoracic	52 (48.1%)
Lumbar	35 (32.4%)
Cervicothoracic	1 (0.9%)
Thoracolumbar	7 (6.5%)
Nature of Durotomy	
Unintentional	55 (50.9%)
Intentional	53 (49.1%)
Method of Dural Repair	
Suture	56 (51.9%)
Patch	45 (41.7%)
Fibrin Glue Alone	7 (6.5%)
Method of Skin Closure	
Dermabond	57 (52.8%)
Suture	8 (7.4%)
Staples	43 (39.8%)

The HOB was flat in 68 out of 108 cases (63%) for a median of three days, with this cohort displaying a median hospital length of stay of six days (Table 4). The failure rate of subfascial CSF diversion was 4.6%, defined as the need for additional procedures such as insertion of a lumbar drain or a return to the operating room (OR) due to persistent CSF leakage or CSF leak-related complications. Of these cases of bile bag failure, four (3.7%) required placement of a post-operative lumbar drain, with only two patients (1.9%) requiring a return to the OR. One patient with persistent incisional CSF leakage required both post-operative lumbar drain placement as well as a return to the OR due to eventual lumbar drain failure. The wound infection rate was 2.8%, with all three cases displaying only superficial wound infections that were successfully managed with antibiotics alone.

**Table 4:** Post-operative characteristics for patients managed with passive subfascial bile bag for durotomy in spine surgery * Total number of patients who required a return to the OR and/or lumbar drain placement; HOB: head of bed; CSF: cerebrospinal fluid

Outcome (N = 108)		
Patient HOB Flat, n (%)		Yes: 68 (63%)
		No: 40 (37%)
Duration HOB Flat (Days), median (IQR)	3 (0-5)	
Hospital Length of Stay (Days), median (IQR)	6 (5-8)	
Bile Bag Failure Rate, n (%)	5 (4.6%)*	
CSF Leak Requiring Post-operative Lumbar Drain, n (%)	4 (3.7%)	
Symptomatic Pseudomeningocele, n (%)	1 (0.9%)	
Persistent Incisional CSF Leak, n (%)	1 (0.9%)	
Post-operative Leak Requiring Surgery, n (%)	2 (1.9%)	
Post-operative Wound Infection, n (%)	3 (2.8%)	

In terms of total length of time with HOB flat based on operated spinal level, patients who underwent spine surgery at or below T7 experienced more days with their HOB flat at a median of four days compared to those who were operated on levels above T7 at a median of 0 days (Table 5). Patients who underwent spine surgery above T7 experienced a similar duration with the subfascial bile bag present compared to those who were operated on levels at or below T7, with both having a median of four days (Table 5).

**Table 5:** Days of HOB flat and bile bag present in relation to operated spine level HOB: head of bed

	At/Below T7/8 (n = 69)	Above T7 (n = 39)	p-value
HOB Flat (Days) (median ± IQR)	4 ± 2	0 ± 1	<0.001
Bile Bag Present (Days) (median ± IQR)	4 ± 2	4 ± 2	0.35

A significant difference in the time spent with a HOB flat and with a subfascial bile bag was found when comparing intentional and unintentional durotomies. Those with intentional durotomies experienced less time with their HOB flat at a median of one day compared to patients who sustained unintentional durotomies at a median of five days (Table 6). The subfascial bile bag was also present longer in those with unintentional durotomies at a median of five days compared to patients with intentional durotomies at a median of four days. The differences in both bile bag and HOB flat duration did not remain significant when comparing intentional and unintentional durotomies for surgery at or below T7, however.

**Table 6:** Days of HOB flat and bile bag present in relation to the nature of durotomy HOB: head of bed

	Intentional Durotomy (n = 53)	Unintentional Durotomy (n = 55)	p-value
HOB Flat (Days) (median ± IQR)	1 ± 4	5 ± 2	0.02
Bile Bag Present (Days) (median ± IQR)	4 ± 2	5 ± 2	0.003

Bile bag success, defined as not requiring a return to the OR or the need for additional procedures to manage a post-operative CSF leak, occurred in 103 (95.4%) patients. In terms of the various skin closure methods utilized within the study population, the usage of staples and Dermabond displayed the lowest bile bag failure rates at only 2.3% and 3.5%, respectively, compared to patients who underwent skin closure via traditional suturing at 25% (Table 7). In terms of the method of dural closure, suture repair displayed the lowest failure rate at only 1.8% compared to closure by muscle or dural patch at 6.7% or by fibrin glue alone at 14.3%. Though the differences in bile bag success between the various skin closure methods were statistically significant (p = 0.02), there was no statistically significant difference between the types of dural closure (p = 0.23). No continuous or categorical variable, including the nature of the durotomy, reached statistical significance between bile bag success and failure.

**Table 7:** Bile bag success by method of dural and skin closure

Type of Closure and Method	Bile Bag Success	Bile Bag Failure	p-value
Skin Closure	n (%)	n (%)	0.02
Dermabond (N = 57)	55 (96.5)	2 (3.5)	
Sutures (N = 8)	6 (75)	2 (25)	
Staples (N = 43)	42 (97.7)	1 (2.3)	
Dural Closure	n (%)	n (%)	0.23
Suture (N = 56)	55 (98.2)	1 (1.8)	
Patch (N = 45)	42 (93.3)	3 (6.7)	
Fibrin Glue Alone (n = 7)	6 (85.7)	1 (14.3)	

Comparing the duration of HOB flat and bile bag drainage across different methods of skin closure did not reveal a significant difference. The method of dural repair, however, revealed significant differences in HOB flat and bile bag duration. Dural closure with patch versus suture alone demonstrated a longer duration of both HOB flat (median four vs three days, p = 0.05) and bile bag duration (median five vs four days, p = 0.006) in the patch group. There were no differences between intentional or unintentional durotomy and skin closure technique; however, there was a significant difference between dural closure and type of durotomy. Suture repair alone was utilized in 86.8% of intentional durotomies, whereas 76.4% of unintentional durotomies were mostly repaired with a patch (p <0.001).

## Discussion

In the present study, we demonstrated that the use of a passive subfascial bile bag for diversion of CSF post-operatively in concert with appropriate HOB management is a safe and effective approach in reducing post-operative symptomatic CSF leak-related complications such as dural cutaneous CSF fistulas, meningeal pseudocysts, and symptomatic pseudomeningoceles [8,11-15]. The T7/8 level is generally where we consider whether the patient should be flat or elevated in bed. While this level is somewhat arbitrary, it approximates where the wound is in a more dependent position, recognizing that there may be equipoise in the mid or lower thoracic spine. Both HOB positioning and subfascial drainage were used in conjunction in this study with the goal of minimizing hydrostatic pressure on the closure site to allow for adequate fascial healing, which could then tolerate additional hydrostatic pressure applied by CSF in the event of continued leakage through the dura.

This management was successful across all represented patient sociodemographics, regardless of comorbid conditions. As noted above, bile bag failure was defined as the need for additional procedures, such as the insertion of a lumbar drain, or a return to the OR due to persistent or complicated CSF leakage. With that in mind, only four patients required post-operative lumbar drain placement for persistent CSF leaks, with only one of these patients requiring a return to the OR. Another patient displayed a symptomatic pseudomeningocele which also required a return to the OR. Of these two cases, however, it should be noted that one of these patients had a diagnosis of Marfan syndrome and was operated on for a symptomatic Tarlov cyst.

The method was also successful whether the durotomy was intentional or unintentional. Although four out of the five cases of drain failure were in the unintentional durotomy group, this failed to meet statistical significance, presumably due to a lack of statistical power. Upon further analysis, the HOB flat and subfascial drain durations were found to be significantly shorter in the intentional durotomy group. Overall, the subfascial drain serves to alert the surgeon whether the dural repair remains functionally intact. Therefore, minimal drain output affords the surgeon to feel confident in both mobilizing the patient and removing the drain earlier. In general, we removed the drain when the overall output tapered off significantly to less than 40cc of drained CSF over a 24-hour period, or after four full days of bedrest regardless of output volume.

There are several important distinctions to be made between our investigation and other similar studies. Niu et. al noted that active suction drains are associated with the development of CSF cutaneous fistulas, pseudomeningoceles, intracranial subdural hematomas, and even the worsening of the CSF leak itself [18]. These issues can be minimized by utilizing a passive or “gravity” drain, such as a bile bag. Still, spine surgeons should be aware that the bile bag can still siphon excessive amounts of CSF if the drain is at a significantly lower level than the leak site, akin to external ventricular drains.

Anderson and Popov investigated the outcome of patients with unintentional lumbar dural tears following suture patch repair and immediate post-operative mobilization. While this study reported only one wound infection and no patients requiring reoperation, they bolstered the primary dural repair with a patch integrated into the primary repair [19]. Hughes et al. demonstrated successful management with prolonged suction drainage continued in the outpatient setting. Although none of their 16 patients with durotomies experienced a complication, there remains a theoretical risk of over-drainage [20]. Another study by Mammadkhanli et al. investigated non-suction drains and involved clamping and intermittent opening of the drain in order to relieve pressure on the fascial closure [21]. Overall, our results and modus operandi are akin to the study by Fang et al. whereby subfascial non-suction drainage is performed without clamping [22]. Decreasing hydrostatic pressure on the fascial closure affords time for this dural and fascial closure to strengthen and withstand the later pressure imposed on it in the event that CSF continues to leak through the dura. We avoid clamping for this reason, which allows the fascia to heal virtually unchallenged by pressurized CSF. In our cohort, we were also able to show a slightly shorter duration of drainage with a high rate of success with neither empiric antibiotics nor acetazolamide routinely utilized.

There are some limitations to be acknowledged with our investigation. First, the small sample size limits the statistical power of the study, and additional research is needed to confirm the efficacy of this technique. Additionally, as decisions concerning the durations of HOB flat and bile bag drainage were surgeon-driven, selection bias was present in our study, and thus our overall findings were likely the result of these decisions rather than the intervention itself. As this was a retrospective investigation limited to surgeries performed by a single surgeon, such findings may not be generalizable to similar patients managed by the same protocol at other institutions. Due to this, only correlations and not causation can be concluded from this investigation. Finally, due to differences in sociodemographic characteristics, spinal pathology, and surgical procedures performed within this study, a notable level of variability was present in our patient cohort. Nonetheless, we find the results of this investigation to be convincing despite these limitations. Future studies are needed to confirm our results across the breadth of spinal pathologies and various types of surgery.

## Conclusions

Intra-operative durotomies, whether intentional or unintentional, are associated with sequelae of long-term post-operative complications, increased financial costs, decreased quality of life in patients, and high rates of subsequent reoperations. Though recommendations such as suture repair are currently being utilized to combat this frequently reported complication, they do not come without flaws. The use of subfascial passive bile bag drainage with a standardized post-operative HOB protocol is an effective technique to reduce post-operative complications and rates of reoperation in patients with intra-operative durotomies. In a sample of 108 patients, the overall rate of return to the OR for true CSF leak-related complications was low at only two patients (1.9%).
